# Chronotherapy in Glioblastoma: State of the art and future perspectives

**DOI:** 10.1016/j.ebiom.2023.104470

**Published:** 2023-02-14

**Authors:** Marina Petković, Melad Henis, Oliver Heese, Angela Relógio

**Affiliations:** a Institute for Theoretical Biology (ITB), Charité—Universitätsmedizin Berlin, Corporate Member of Freie Universität Berlin, Humboldt-Universität zu Berlin and Berlin Institute of Health, Berlin 10117, Germany; b Institute for Systems Medicine and Faculty of Human Medicine, MSH Medical School Hamburg, Hamburg 20457, Germany; c Department of Neurosurgery and Spinal Surgery, HELIOS Medical Center Schwerin, University Campus of MSH Medical School Hamburg, Hamburg 20457, Germany; d Medical Department of Hematology, Oncology, and Tumour Immunology, Molecular Cancer Research Center (MKFZ), Charité—Universitätsmedizin Berlin, Corporate Member of Freie Universität Berlin, Humboldt-Universität zu Berlin and Berlin Institute of Health, Berlin 10117, Germany

**Keywords:** Chronotherapy, Circadian medicine, Circadian rhythm, Glioma, Glioblastoma, Brain cancer

## Abstract

Circadian rhythms regulate various processes in the human body, including drug metabolism. Chronotherapy optimizes treatment timing based on the circadian rhythm of the individual patient, such that the treatment efficacy is maximized, and adverse effects are minimized. It has been explored in different cancers with varying conclusions. Glioblastoma multiforme (GBM) is the most aggressive type of brain tumour with a very dismal prognosis. In recent years, there has been very little success in designing successful therapies to fight this disease. Chronotherapy offers the opportunity to leverage existing treatments to extend patient survival and to increase their quality of life. Here, we discuss recent advances in using chronotherapy regimens in the treatment of GMB, such as radiotherapy, temozolomide (TMZ) and bortezomib, as well as discuss novel treatments with drugs of short half-life or circadian phase specific activity, and examine the therapeutic potential of new approaches that target elements of the core circadian clock.

## Introduction

Circadian clocks, driving daily rhythms, regulate various processes in the human body. Circadian rhythms (CR) are generated by an endogenous biological clock and control the timing of physiology and behaviour in mammals, e.g., the 24-h sleep–wake cycle, in synchrony with the environmental intervals of light and darkness. A robust circadian clock is crucial for maintaining cellular homeostasis. Its dysregulation has been associated with a plethora of pathologies, including sleep disorders,^1^ mental disorders,^2^ neurogenerative diseases,^3^ and cancer.^4^ In mammals, the circadian clock regulates the rhythmic expression of approximately 40% of all protein-coding genes across different tissues,^5^ resulting in the regulation of numerous biological processes such as RNA processing,^6^ metabolism,^7^ cell cycle,^8^ apoptosis,^9^^,^^10^ immune system^11^ and even cellular migration and metastasis.^10^

Circadian rhythms in all body cells are driven by a transcriptional/translational molecular network of activators and repressors that operate in interlocking feedback loops.^12^ The core-clock proteins, aryl hydrocarbon receptor nuclear translocator-like protein 1 (ARNTL, also known as BMAL1) and circadian locomotor output cycles kaput (CLOCK) form heterodimers, bind to E-box elements in the promoter region of the genes period (*PER1, 2, 3*) and cryptochrome (*CRY1, 2*) and initiate their transcription. Once in the nucleus, PER/CRY heterodimers prevent binding of the ARNTL/CLOCK complex to E-boxes and inhibit the transcription of ARNTL/CLOCK target genes. In addition, ARNTL/CLOCK heterodimers activate the transcription of Nuclear Receptor Subfamily 1 Group D Members (REV-ER α, β) and Retinoic Acid Receptor-Related Orphan Receptors (ROR α, β, γ). REV-ERBs and RORs in turn compete for ROREs binding sites in the promoter region of ARNTL and fine tune its transcription via inhibition (REV-ERBs) or activation (RORs), respectively. These core-clock elements regulate the circadian expression of a plethora of genes in different tissues.^13^^,^^14^ The disruption of the rhythmic expression of core-clock and clock-regulated genes is associated with various cancers, including glioblastoma multiforme (GBM).^15^

**Fig. 1:**
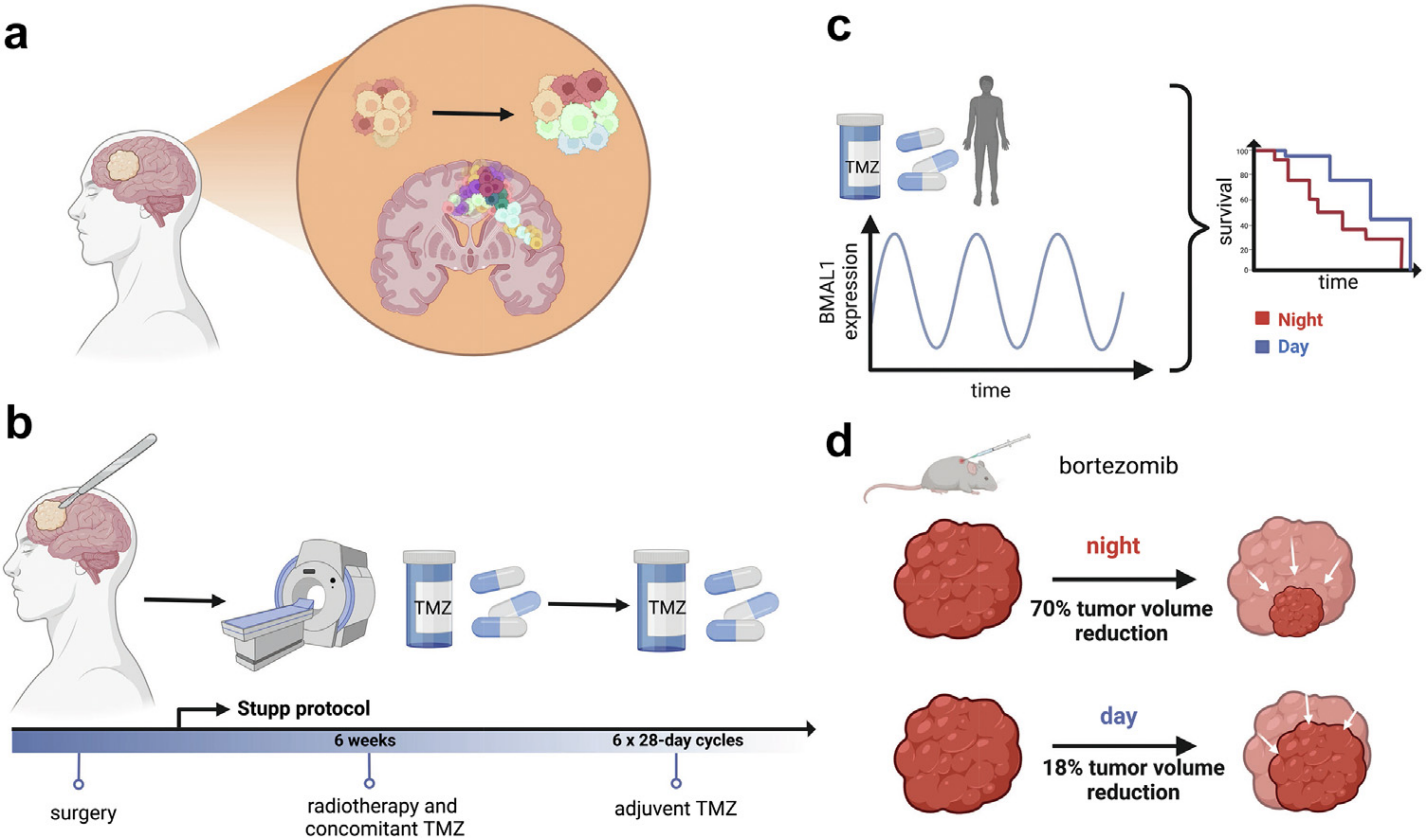
**Glioblastoma overview and treatment options.** a) GBM displays high intra-tumour heterogeneity and an infiltrative nature. b) Stupp protocol for GBM treatment. c) Chronotherapy regimen for TMZ in humans. Morning administration of TMZ led to longer OS vs evening administration (median OS, 95% confidence interval [CI] = 1.43, 1.12–1.92 vs 1.13, 0.84–1.58 years).^18^ d) Chronotherapy regimen for bortezomib in mice. Treatment at the beginning of the night (ZT 12–13), using low dosage concentration of the drug, led to 70% tumour growth inhibition, vs the same concentration during the beginning of the day (ZT 1–2), which led to 18% growth inhibition.^19^

GBM is one of the most aggressive and highly invasive brain tumours (Fig. 1a) that originates from astrocytic or oligodendrocytic glial cells.^16^ Due to its pronounced mitotic activity and following mass expansion patients with GBMs have a short period of aggravating symptoms over a couple of weeks, sometimes days like headache, weakness, seizure, memory disturbances, visual and speech abnormalities. The subsequently increasing intracranial pressure results in loss of consciousness and finally, if not treated adequately in death.^17^ This tumour originates mainly as a primary tumour with malignant features (90% of cases diagnosed with GBMs) and is classified as grade WHO IV, while in 10% of the cases it develops as secondary tumour arising from a previously diagnosed low grade astrocytoma or oligodendroglioma WHO II or WHO III. In this review, we followed the former WHO classification of gliomas, indicated using Roman numbers I–IV, to align with the studies considered for the review, where patients were stratified according to this historic classification, and not according to the molecular parameters in the revised WHO classification (1–4) from 2021.^20^ On a molecular level primary GBMs are distinguished from secondary GBMs by IDH-1 mutation status.^21^ GBM accounts for 55% of malignant brain tumours in adults, with the incidence between 3.19 and 4.17 new cases per 100,000 persons per year.^22^ GBM occurs most frequently in older adults (60–70 years old), and is more commonly diagnosed in males than in females.^23^ Treatment options for GBM include the Stupp standard care protocol^24^ that combines surgical tumour resection followed by radio and chemotherapy with temozolomide (TMZ), with subsequent multiple rounds of adjuvant TMZ treatment (Fig. 1b), as well as tumour treating fields (TTF) therapy.^25^ TTF are evaluated in a phase III trial (EF-14) as first line treatment following the 6 weeks radiochemotherapy and then combined to cyclic TMZ. So far TTF as second line treatment is neither re-imbursed by insurance companies in several countries nor is it proven to be effective in phase III studies (negative trial EF-11). In patients with a MGMT-methylation (O-6-Methylguanine-DNA Methyltransferase methylation) status in the tumour tissue, intensified chemotherapy protocols with addition of lomustine (Chlorethyl-Cyclohexyl-Nitroso-Urea, CCNU) can be applied. From the neurosurgical point of view the gross total resection without inducing new neurological deficits is the primary goal, which means resection of the contrast-enhancing tumour according to MRI criteria. Due to the specific tumour biology with the highly infiltrative features and tumour cell migration into the surrounding functional brain tissue a biological complete resection is not possible. Radiation therapy of the tumour surrounding tissue is fractionated in 30 doses of 1.8Gy–2Gy for the total dose of 54Gy–60Gy. Median survival of patients is 12-15 months, and the tumour recurs after the initial treatment in 90% cases locally.^26^ There are multiple reasons for the lack of successful treatment options for GBM, including a high degree of intra-tumour heterogeneity (Fig. 1a), which makes it difficult to develop targeted therapies. In addition, the blood–brain barrier (BBB) poses another challenge for delivering chemotherapeutics to the brain. Moreover, tumour microenvironment of GBM promotes resistance of the tumour to chemotherapy and radiotherapy, and the tumour shows a low immunogenicity, which prevents a proper immune response.^27^ Corticosteroids are the most common drug compromising immune responses in glioma during and after treatment. Due to their potent role in reducing interstitial edema corticosteroids are used to relief symptom burden in particular during radiochemotherapy.

Considering the very short overall survival (OS) of GBM patients and the lack of successful local and systemic treatments, new strategies are needed to improve the treatment efficacy, diminish its side effects, and improve life quality of patients.

Nowadays, chronotherapy is considered as an emerging strategy in treatment of cancer.^28^ It is based on precisely timing administration of treatments based on the patient's circadian rhythm to maximize the treatment efficiency, while minimizing its side effects. Chronotherapy was explored in melanoma,^29^ colorectal,^30^^,^^31^ ovarian cancer,^32^^,^^33^ breast cancer,^34^^,^^35^ non-small-cell lung carcinoma,^36^ nasopharyngeal carcinoma,^37^^,^^38^ and glioblastoma^18^^,^^39^ with varying conclusions. Time-dependent administration of treatment in patients with non-small-cell lung carcinoma,^36^ melanoma^29^ and rats with ovarian cancer^40^ prolonged their survival. However, in patients with colorectal cancer, the timed drug administration improved outcomes only of males.^31^ Patients with nasopharyngeal carcinoma who received chronotherapy regimen experienced reduced side effects.^38^ In addition to the previous anti-cancer interventions, chronotherapy has been applied to various pathologies e.g., asthma,^41^ cardiovascular diseases^42^ and hypertension,^43^ which led to a general improvement of patient's condition.

Circadian rhythms play a crucial role in the pharmacodynamic (biochemical, molecular and physiological effects in the organism) processes of the therapeutic agents.^44^ Accordingly, the lethal toxic dose of a therapeutic varies during the day, as demonstrated in experiments on mice and rats.^45^ Moreover, 24 h variations have been observed in processes that dictate drug disposition, such as absorption, distribution, metabolism, and elimination.^44^ Many cancer therapeutics are cytotoxic in different phases of the cell cycle.^45^ For example, cells in S-phase (DNA synthesis phase) are more responsive to 5-fluorouracil (5-FU) and irinotecan, frequently used to treat colorectal cancer.^45^ 5-FU inhibits thymidylate synthase, which disrupts the synthesis of pyrimidine thymidylate required for the DNA replication.^46^ Furthermore, it inhibits RNA synthesis by integrating its metabolites into RNA.^46^ On the other hand, irinotecan interacts with topoisomerase I leading to inhibition of DNA synthesis with double-strand DNA breaks and cell cycle arrest resulting in cell death.^47^

Both computational models based on cell cycle states or those based on the dynamics transcriptional/translational clock networks and of relevant drug-related metabolic pathways, and clinical studies showed that the timed drug administration impacts normal and tumour cells differently and according to their internal circadian clock.^48^ Such a disparity is observed because clock and clock-regulated genes in tumour cells frequently have different circadian profiles (either shifted or completely disrupted), or even different period lengths,^49^, ^50^, ^51^, ^52^ which allows to choose the optimal time at which the drug is more cytotoxic for tumour cells, as compared to the healthy cells in the organism. Furthermore, the cytotoxicity of anticancer drugs is determined by their metabolism and detoxification, as well as cell-cycle related targets, apoptosis and DNA repair, which are all controlled by the circadian clock.^45^ In the treatment of colorectal cancer, 5-FU is combined with oxaliplatin and/or irinotecan. Oxaliplatin, which induces cell cycle arrest and apoptosis,^53^ was previously rejected for the treatment of colorectal cancer due to its excessive toxicity and poor activity in the disease.^54^ However, chronomodulated administration of irinotecan, oxaliplatin, 5-FU and leucovorin (which enhances 5-FU activity) improved survival and reduced the adverse effects of drugs in patients with metastatic colorectal cancer.^55^ Recent studies showed evidence of the efficacy of a timed radiotherapy, specifically in adenocarcinoma. Patients who received radiotherapy in the morning had reduced side effects.^48^ Additionally, gender plays a role in the temporal response to the radiotherapy. Females who received radiotherapy in a 11AM–2PM interval had a significantly better response than males.^48^ Interestingly, irinotecan has a sex-dependent least toxic timing. While its administration in the morning during treatment of metastatic colorectal cancer reduced the adverse effects in males, its afternoon administration in females has minimal side effects.^56^ Standard therapies for patients with advanced ovarian cancer include doxorubicin and cisplatin.^57^ Administration of these medications in a timed manner, with doxorubicin given in the morning and cisplatin in the evening, exerted reduced renal toxicity with fewer complications compared to their administration at different times of day.^33^

Overall chronotherapy studies so far point to an improvement in terms of side-effects of anticancer treatment and either no changes or also an improvement in survival^28^ making chronotherapy a promising new approach in the treatment of cancer.

GBM is a very aggressive disease with poor prognosis, despite efforts in transferring new drug developments and therapies from other cancer entities to multimodal treatments of GBMs, so far numerous phase III studies lacked showing an effect in prolonging overall survival (OS) or progression-free survival (PFS) in these patients. Moreover, the design of new therapies for GBM poses a challenge due to its high heterogeneity, its infiltrative nature and the challenge of the BBB permeability. Therefore, exploring different administration schedules of already existing treatments in chronotherapy that either extend survival or mitigate the side effects is worth exploring. Moreover, strategies to target the elements of circadian clock to restore the circadian rhythmicity are becoming more interesting. In this review, we provide a synthesis of current studies that explore chronotherapeutic strategies in GBM and describe drugs targeting circadian clock elements. In particular, we will address the recent advances in chronomodulated application of radiotherapy, TMZ and bortezomib in human and mouse studies, as well as potential therapeutics that could be included in the future trial of GBM chronotherapy. Considering the relevance of a dysregulated circadian clock in the development of gliomas and its clinical research, we will review potential anti-GBM agents that target the clock molecular machinery in GBM patients, and that may be applied in the future chronotherapy regimes for GBM. Finally, we will discuss the need for circadian considerations in future GBM studies mainly given the increasing amount of knowledge in the circadian field with direct applications to medicine in particular to anti-cancer treatment the reduction of its side effects and overall improvement of life quality for the patients.

## Chronotherapy drugs/approaches in glioblastoma

### Standard treatment for glioblastoma

The standard treatment for newly diagnosed GBM follows the Stupp protocol (Fig. 1b), and includes microsurgical gross total resection, followed by the concomitant therapy with radiotherapy and temozolomide (TMZ), and additional adjuvant therapy with TMZ.^24^

Since MGMT-methylation (metyl-guanin-metyl-transferase) predicts the response to chemotherapy with alkylating substances like TMZ, in patients with this specific mutation intensified chemotherapy protocols with the addition of CCNU have shown positive signals in phase III studies.^58^ The side effects of the TMZ and/or CCNU chemotherapy include nausea, and in some patients, severe myelosuppression which leads to the discontinuation of therapy.^24^

**Fig. 2:**
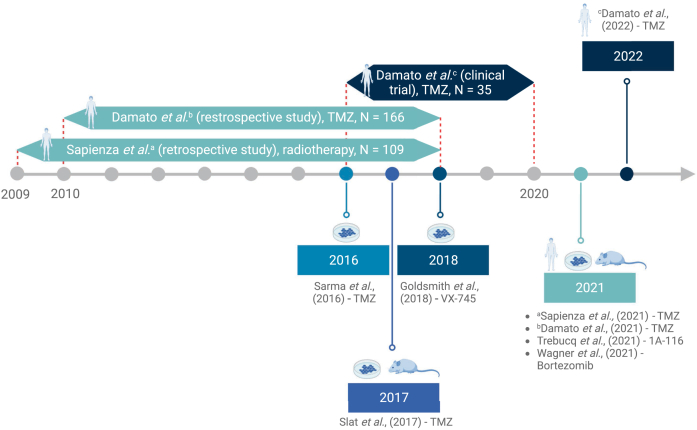
**Timeline of key chronotherapy studies in GBM.** Publication years are highlighted in rectangles. The recruitment time for each of the human studies is indicated with double-sided arrows on the timeline.

Several treatment approaches have recently been tested as part of the chronotherapy regime for the GMB treatment, either on humans, mice and rats, or human cells. These treatments include chronoradiotherapy,^59^ and chemotherapy with TMZ,^39^^,^^60^ bortezomib,^19^ 1A-116^61^ and curcumin.^62^ An overview is provided in Fig. 2 and Table 1.

**Table 1:** Studies with chronotherapy applications for GBM.

Study/reference	Treatment	Experimental model	Participant characteristics	Effect on survival	Effect on side effects
Sapienza et al.^59^; a retrospective study	Radiotherapy	Human; high-grade glioma (grade III and IV), 80% patients with GBM - grade IV	109 (62 males, 47 females), average age = 62.6	No difference in OS and PFS between patients treated in the morning or in the afternoon.	No significant difference in adverse effects between the patients treated in the morning or in the afternoon.
Damato et al.^39^; a randomized feasibility study, clinical trial ID: NCT02781792	TMZ	Human; grade II-IV (60% patients with grade IV - GBM)	35 (15 female, 20 male); average age = 56.31	No significant difference in OS between the morning and the evening administration of TMZ.	No significant difference in adverse effects between the morning and the evening administration of TMZ.
Damato et al.^18^; a retrospective study	TMZ	Human; GBM	166 (61 female, 105 male), average age = 60.1	Morning administration of TMZ in MGMT-methylated patients extends the OS for 3.6 months and for 6 months in MGMT methylated patients.	Data on adverse effects not collected
Slat et al.^60^	TMZ	Mesenchymal GBM astrocytes from male *Nf1*^*flox/flox*^*; GFAP-Cre* mice	/	The highest sensitivity to TMZ at the peak of *Bmal1* expression.	/
Trebucq et al.^61^	1A-116	Human GBM cell line LN229, mouse xenografts on male NIH Swiss foxN1 (Δ/Δ) nude mice	/	Extended survival upon the evening drug application. The application of low concentration of 1A-116 near the peak of TIAM1 expression gives similar effects of applying saturating concentrations at different time points.	The study did not explore the impact of chronotherapy on adverse effects in LN229 mouse xenografts.
Wagner et al.^19^	Bortezomib	Synchronized glioma cells A530 injected into male C57BL/6 mice	/	Higher efficacy of the low-dose Bortezomib administered at night.	Chronotherapy regimen with low doses of bortezomib mitigates the side effect of the 12%–20% weight loss that coincided with high doses
Sarma et al.^62^	curcumin	GBM; rat C6 glioma cell line		Low dose of curcumin increases cell death days after the administration. Curcumin caused persistent cell death well after it was detectable in the medium.	/
Goldsmith et al.^63^	VX-745	GBM; cell lines – HA (human astroglia), IM3, C6, mouse Per2^Luc^ SCN cells and fibroblasts, mouse Bmal1-dLuc fibroblasts, mouse Per1^ldc^/Per2^ldc^ SCN cells and fibroblasts	/	Application of VX-745, a p38 MAPK inhibitor, reduces its activity and reduces cell invasiveness	/

### Chronoradiotherapy

Radiation therapy is part of the standard treatment for GBM following surgical resection. Moreover, scheduling radiotherapy treatments based on time of the day in rectal cancer (morning vs afternoon),^64^ prostate cancer (daytime vs evening)^65^ and bone metastasis (8AM–11AM vs 11AM–2PM vs 2PM–5PM)^66^ showed benefits in maximizing the effect of radiotherapy and/or in minimizing its side effects. Recently, Sapienza and colleagues^59^ performed a retrospective analysis on 109 GBM patients (62 males, 47 females) who received radiotherapy treatment either in the morning or in the afternoon. The authors found no significant differences in progression-free survival (PFS), overall survival (OS), and treatment toxicity between the two treated groups, which implies that either high-grade gliomas are less responsive to the time-based delivery of the radiotherapy, or that the patients in the different groups (morning vs afternoon) were not treated at their optimal treatment time, according to their circadian clock.

### Temozolomide

Temozolomide (TMZ), alongside radiotherapy, is part of the standard treatment for GBM. Its introduction in the standard treatment regimen extended the OS times of the patients.^24^ Considering that TMZ has a short half-life in plasma (1.8 h)^67^ and it crosses the BBB easily, a few studies have explored administering TMZ based on the circadian rhythm in humans^18^^,^^39^ and mouse GBM cells.^60^ Intriguingly, the sensitivity of human and murine GBM cells to the TMZ-induced DNA damage, apoptosis, and growth inhibition was shown to be the most pronounced around the peak of *BMAL1* expression, that is in the morning for both humans and mice (housed under a 7AM lights-on, 7PM lights-off schedule, mimicking human light exposure).^60^ Yet, the active phase of mice (locomotor activity, metabolism and feeding routines) happens during the lights-off period, and thus considerations due to shifts in the endogenous CR need to be taken into account. Still, these results suggest that the circadian clock can regulate TMZ toxicity in GBM also in humans, and that adapting TMZ administration to the peak of *BMAL1* expression in GBM cells can enhance its efficiency,^60^ (Fig. 1c).

Recently, a retrospective study analysed the difference in OS between the patients who received adjuvant TMZ in the morning on an empty stomach, vs those who received the treatment in the evening. Prior to adjuvant TMZ, all patients underwent radiotherapy with concurrent TMZ in the morning.^18^ This study reported a higher median OS when TMZ was administered in the morning (1.43 years for the AM group, 1.13 years for the PM group), during a 6 h time window around the peak of BMAL1 protein expression. Given the mechanism of activity of TMZ, this points to a role of BMAL1 in the cell's response to DNA damage. Moreover, some cells can repair the damage and avoid cell death by expressing the O-6-Methylguanine-DNA Methyltransferase (*MGMT*) gene. Such cells are generally resistant to the TMZ treatment. Following this notion, morning administration of TMZ to the subgroup of patients with methylated MGMT increased the OS up to 6 months.^18^

Currently, a phase 2 randomized clinical trial evaluating TMZ chronotherapy for high grade glioma (NCT02781792) is in progress.^68^ The trial, evaluating 39 patients, tests the effect of administration of TMZ in the morning (before 10 AM) vs in the evening (after 8 PM) on the patient survival and quality of their life. The preliminary results indicate no significant difference in the OS between the morning and evening administration of TMZ for patients with glioma II–IV.^39^ Furthermore, adverse effects were also not significantly different between the two groups, although the group with the morning administration reported 3 haematological treatment-emergent adverse effects of grade 3 or higher, which were absent in the evening group.^39^

### 1A-116

Another therapeutic that has recently been explored for the treatment of GBM is 1A-116, which has a proapoptotic and anti-invasive activity in malignant glioma, and has been reported as a promissing candidate for conventional therapy^69^ and as chronotherapy.^61^ Mechanistically, 1A-116 inhibits Ras-related C3 botulinum toxin substrate 1 (RAC1) GTPase in human glioma cells and mice.^69^ RAC1 a master regulator of cell motility^70^ involved in various cellular processes necessary for tumour development, such as proliferation, migration, invasion, angiogenesis, and cytoskeletal organization.^71^ Furthermore, 1A-116 inhibits Rac1 signalling pathway by preventing its interaction with T-lymphoma invasion and metastasis-inducing protein-1 (TIAM1), a guanine exchange factor,^61^ whose high expression is associated with increased lymphatic metastasis and worse patient survival in different cancers.^72^ Additionally, 1A-116 has a chronotherapeutic effect against GBM and its efficacy is regulated by the circadian clock. In a study on mouse xenografts (with GBM LN229 cells), Trebucq et al^61^ reported that low concentrations of 1A-116 applied near the peak of TIAM1 expression have similar effects for its application with saturating concentrations at different time points. Interestingly, the survival of animals bearing the xenografted tumour was extended when the drug was applied in the evening compared to daytime, which suggests chrono-modulation administration of 1A-116 enhances GBM treatment.^61^

### Bortezomib

Bortezomib is a proteasome inhibitor used in the treatment of multiple myeloma^73^ and cell mantle lymphoma.^74^ Bortezomib was recently investigated as a candidate for chronotherapy using A530 glioma cells (isolated from malignant peripheral nerve sheath tumour of *Trp53*^*+/−*^*; Nf1*^*+/−*^ mice), injected in C57BL/6 mice, as a model for GBM.^19^ The study on mouse GBM cells^19^ showed that the low-dose bortezomib therapy led to higher treatment efficacy when administered at night. Moreover, the reported side effect of a weight loss of 12%–20% body mass when the high dose of bortezomib was administered, was mitigated by administering the low dose of the therapeutic (Fig. 1d). As such, chronomodulated application of bortezomib may enable its anti-cancer effects, while mitigating side effects related to the administration of high doses.

### Drugs with chronotherapeutic potential

A few therapeutics have a potential to be explored in chronotherapy procedures due to their short half-life or due to their circadian phase specific activity. Curcumin, a phytochemical with anti-inflammatory, anti-microbial and wound-healing properties,^62^ has a short half-life, making it a good candidate for a chronotherapy regimen.^62^ It inhibits multiple signal transduction pathways, which results in the inhibition of cellular proliferation and induction of apoptosis. A major advantage in the usage of Curcumin as a therapeutic agent, is its lower toxicity for normal cells, as compared to currently used anti-cancer drugs. Moreover, its administration in combination with cisplatin and doxorubicin, curcumin induces apoptosis in GBM cell lines and in preclinical models of glioblastoma.^75^ In rat C6 glioma cell lines, the application of a low dose of curcumin resulted in increased cell death.^62^ However, the higher dose of curcumin resulted in the loss of detectable circadian rhythms in apoptosis, but the circadian rhythm in mitotic events was not lost. This dose was still lower than the IC50 value of curcumin in C6 cells.^62^ Moreover, increased cell death was observed long after curcumin was detectable in the medium. The authors mention several possible explanations for such an effect. The first being that Curcumin could have caused mitotic arrest, resulting in a delayed cell death, or it could have caused epigenetic changes, which promote cancer cell death. An alternative explanation could be that Curcumin may have remained trapped in the cell membrane and released at a later stage. Finally, it is also conceivable that a delayed cell death could have been caused by curcumin congeners with anti-cancer properties, which persist much longer in the medium. These results point to a possible role of curcumin as an anti-cancer drug.^62^ Still, further research is needed to establish the specificity of curcumin, as well as the effect of different curcumin concentrations on the rhythmicity in apoptotic and mitotic events.

VX-745 is an inhibitor of p38 mitogen activated protein kinase (p38 MAPK), a potential oncogene factor in glioma.^63^^,^^76^ Phosphorylation of p38 MAPK activates the p38 MAPK pathway, and regulates stress responses, such as proliferation, differentiation, development, and apoptosis. The connection between circadian clock and p38 MAPK pathway has been shown in different *in vivo* and *in vitro* models, such as *N. crassa*,^77^ rat pineal gland,^78^ hamster SCN^79^ and mouse heart,^80^ as well as mouse fibroblast and mouse SCN cell lines.^63^ In glioma cells, p38 MAPK activity was found to be arrhythmic, with expression levels in the highly invasive IM3 comparably higher than in C6.^63^ Moreover, application of VX-745 when the levels of phosphorylated p38 MAPK are the lowest lead to the decrease in cell invasiveness.^63^

Due to their specific characteristics, with curcumin having a short half-life and VX-745 being a potential target for restoring rhythmicity in glioma cells, these compounds might be more efficient when administered at specific times and may in the future be explored as chronotherapeutic agents.

## Elements of clock as therapeutic targets

As mentioned above, the circadian system has been associated with different physiological and pathological cellular processes. Several lines of published evidence support the strong connections between the dysregulated circadian clock and clock-controlled genes with cancer development and its progression. This theory is supported by a comprehensive bioinformatic study that revealed alterations of the clock genes across multiple human cancers, which highlights the clinical importance of targeting the core-clock network in cancer chronotherapy.^81^ In gliomas, clock genes show differential expression patterns that affect their molecular pathogenesis. An analysis on data from The Cancer Genome Atlas (TCGA) database showed an upregulation of *BMAL1* in high-grade glioma patients,^82^ while *BMAL1* was downregulated in patient-derived glioma stem cells (GSC) and impaired their progression.^83^ Accordingly, the downregulation of *BMAL1* or *CLOCK* in GSCs induced cell-cycle arrest and apoptosis through attenuation of mitochondrial metabolic function and reduced expression of tricarboxylic acid (TCA) cycle enzymes.^82^ On the other hand, recent studies suggested that the gain of function of *BMAL1* via adenovirus-mediated expression of *BMAL1* reduced proliferation, migration, and invasion of U87MG glioblastoma cells due to the downregulation of p-AKT and Metalloproteinase-9 pathways,^84^ while its downregulation is associated with high proliferation rates and aggressiveness of the tumour.^19^ Thus, while in one study *BMAL1* knockdown was reported to induced cell-cycle arrest and apoptosis,^82^ in a different study *BMAL1* overexpression reduced glioma invasiveness, rather pointing to *BMAL1* as a potential tumour suppressor.^85^

Furthermore, CLOCK is involved in the proliferation and migration of glioma cells from high-grade human glioma tissues and GBM cell lines, through the activation of NF-kB signalling pathway^86^ and CLOCK is downregulated in GBM samples.^87^ Furthermore, a recent study combining genomic, transcriptomic and clinical data analysis of different cancer types revealed the association of downregulation of *CLOCK* genes with higher mortality of glioma patients.^88^ It is noteworthy that the expression of *PER1*, *PER2*, *PER3* is also downregulated in high-grade gliomas and associated with increased proliferation and survival of glioma cells.^87^ These studies suggest that the *PER* genes, specially *PER1*, *PER2*, are critical to GBM formation and act as tumour suppressor genes.^49^

Based on the results described above together with the recently reported data,^89^ future targeting of *BMAL1*, *CLOCK* or genes from the *PER* family may represent promising therapeutic targets for treatment of glioblastoma.

**Fig. 3:**
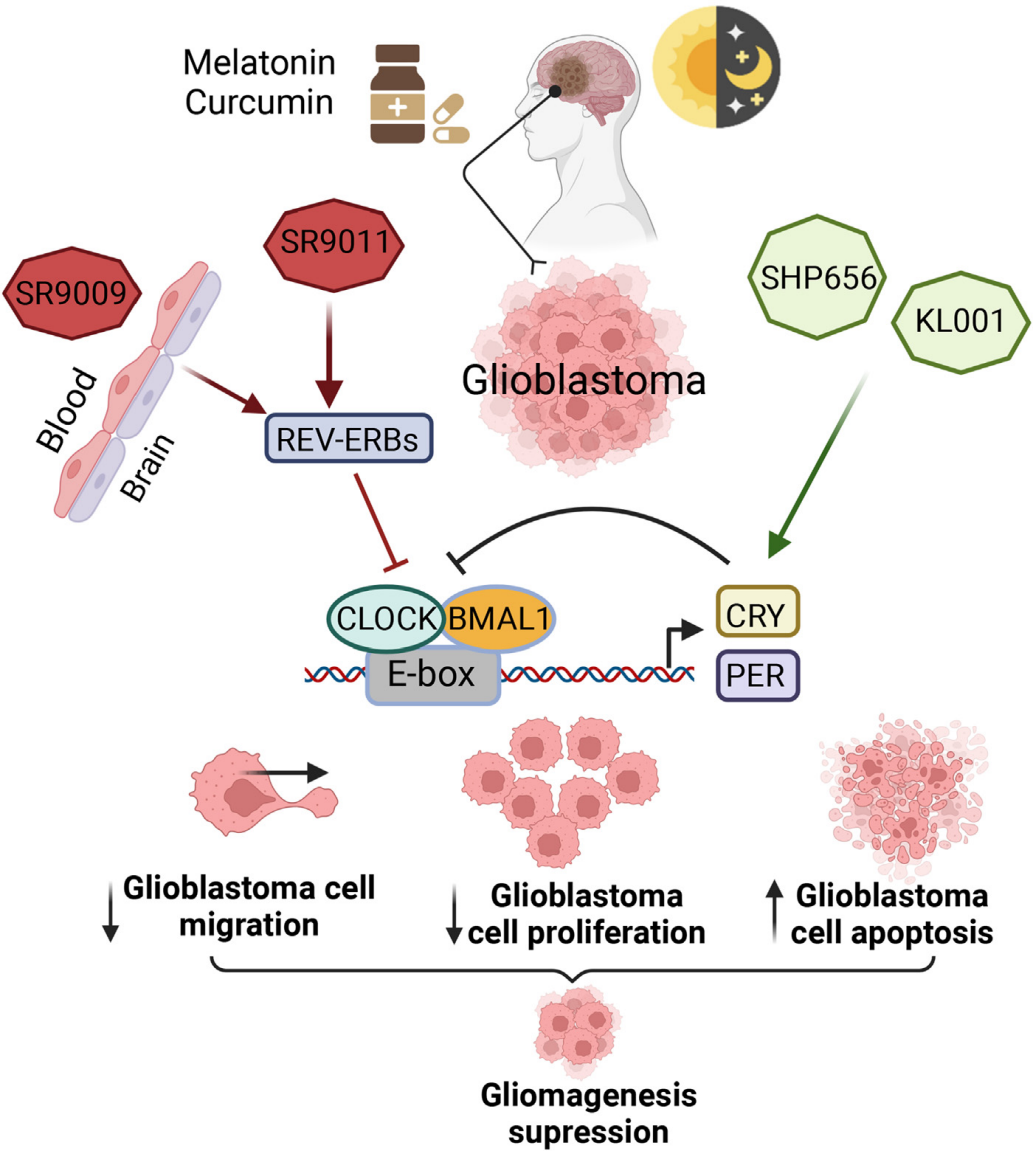
**Targeting of circadian clock components suppresses GBM development.** Circadian rhythm regulation, and natural (melatonin, curcumin) and synthetic compounds orchestrate to decrease cell migration, differentiation and induce apoptosis in glioblastoma cells. KL001 and SHP656 stabilize CRY proteins, which prevent binding of the BMAL1/CLOCK complex to E-boxes inhibiting the transcription of BMAL1/CLOCK target genes. SR9011 and SR9009 are agonists of REV-ERB, an inhibitor of *BMAL1* transcription.

In the past 5 years, a growing number of preclinical studies to pharmacologically target the circadian clock in the treatment of circadian disruption-associated diseases have delivered motivating results in cancer therapy.^90^ One such study focuses on the treatment of GBM based on the modulation of CRY proteins by KL001 and its derivative SHP656, synthetic agonist that stabilizes CRY1/2 levels. KL001 anti-tumour properties in GBM stem cells have been previously described, and its administration results in decreased cellular migration and proliferation and increased apoptosis in GSCs.^82^ Recently, it has been reported that SHP656 lengthened the cellular circadian period in a CRY2-dependent manner in human U2OS cells, harbouring either a *BMAL1* promoter–luciferase (*BMAL1*-dLuc) reporter or a *PER2*-dLuc reporter. These observations suggest that CRY2 is a potential therapeutic target for the treatment of GBM.^91^ Other small synthetic molecules, agonists of the nuclear receptors REV-ERBs (SR9011 and SR9009) that inhibit *Bmal1* transcription under normal conditions, are lethal in different cancers including GBM.^92^ Remarkably, in *in vitro* studies, both SR9011 and KL001 impair the proliferation of the glioma stemness, as well as reduce glioma growth and extend mouse survival in *in vivo* studies.^82^^,^^92^ Interestingly, the efficacy of these pharmacological agents in reducing tumour growth in GBM mouse models was similar to that observed with TMZ in glioblastoma patient-derived xenografts.^92^ These studies highlight that the pharmacological modulation of circadian clock genes can impair cell proliferation, which is a well-known hallmark of gliomagenesis (Fig. 3).

As mentioned in the introduction, light plays a vital role in synchronization of the circadian machinery and cellular homeostasis and regulates melatonin levels. Melatonin is a naturally synthesized hormone and is involved in circadian rhythm regulation via the control of sleep and wake cycles. Pharmacological interventions by different melatonin agonists are widely used in circadian medicine to synchronize circadian rhythms and control sleep disruptions,^2^ and neuropsychiatric disorders.^93^ Melatonin has antiproliferative action and inhibits the growth of multiple types of tumours.^94^ Melatonin reportedly has anti-proliferative action on GBM cells both *in vitro* and *in vivo*, and its administration synergistically increases the effectiveness of the anticancer agents.^94^ A comprehensive study by Gengatharan et al, 2021^95^ using *in vivo* imaging showed that melatonin has an inhibitory effect on the neural stem cell (NSC) proliferation located in the subventricular zone (SVZ). Consistently, the reduction of circadian melatonin synthesis levels and release or of its receptors in the SVZ facilitates GBM initiation and growth.^96^ It was also proposed that melatonin may have a therapeutic role in reducing GBM cell proliferation^97^ and disruption of light-dependent melatonin synthesis, enhanced glioma tumour growth in an *in vivo* study,^98^ highlighting the correlation between melatonin and GBM. Furthermore, melatonin reduces chemotherapeutic drug resistance in GBM stem cells.^99^ These studies suggest the inhibitor role of melatonin in GBM, as well as its effective combination as an adjuvant agent with TMZ and other chemotherapeutic agents during the treatment of glioma to avoid drug resistance and increase their efficacy.

Synthetic or natural anti-glioblastoma compounds such as SR9009 and Bortezomib or Curcumin, respectively, have a temporal anti-cancer activity depending on the circadian metabolism in GBM.^50^^,^^62^^,^^100^ Furthermore, the permeability of the BBB is considered one of the challenges facing the efficient treatment of glioblastoma. BBB hinders the efficiency of drug delivery, thereby limiting the efficient treatment, and only small molecule drugs or genes can pass the BBB.^101^ Reportedly, many previous studies have been conducted aiming to find better strategies to enhance BBB permeability of the BBB and improve glioblastoma drug delivery.^102^ In this regard, curcumin is permeable to the BBB and has a potential therapeutic in Alzheimer's disease,^103^ in addition its analogue C-150 increased the survival rate in treated rats with glioblastoma through NF-κB, UPR and Akt/Notch signalling pathways.^104^ Recently it has been reported that curcumin targets different molecules regulating the circadian timing system with anti-cancer activity such as PER2, BMAL1, and CLOCK.^105^ Moreover, SR9009, which is considered a promising potential agent for GBM treatment,^26^^,^^92^ can easily cross the BBB.^106^ Interestingly, the circadian clock is involved in BBB permeability regulation in mammalian cells,^107^ which highlights the crosstalk between circadian clock regulation and the efficiency of therapeutics delivery to the CNS during the treatment of GBM. Based on the outcomes of the previously mentioned studies, summarized in Table 2, the circadian clock is likely to play a relevant role in the treatment of GBM. While chronotherapy has the potential to maximize the efficiency of the treatment and minimize its side effects,^28^ the efficacy of the therapeutics has also been linked to the expression of specific core-clock genes. It will thus be necessary to consider, on one hand, the precise timing in the administration of anti-glioblastoma therapeutics, and on the other hand, the usage of agents that target clock elements, based on the patient's circadian rhythm.

**Table 2:** Genetic and pharmacological targeting of molecular clock machinery in GBM.

Experimental approach/drug	Experimental model	Potential effect	Potential mechanism	Reference
*BMAL1* knockdown	Human GBM stem cells. Intracranial tumour xenograft- mouse	Suppressed proliferation Cell-Cycle Arrest Induced Apoptosis Reduced tumour size (*in vivo*)	Attenuation of mitochondrial metabolic functions and expression reduction of TCA cycle enzymes in GBM stem cells.	Dong et al.^82^
*BMAL1* knockdown	Human GBM stem cells. Intracranial tumour xenograft- mouse.	Suppressed proliferation Reduced microglia infiltration Reduced tumour growth	Triggered pro-tumour immunity via transcriptional up-regulation of OLFML3, a novel chemokine recruiting immune-suppressive microglia into the tumour microenvironment	Chen et al.^83^
*CLOCK* Knockdown	Human GSCs. Intracranial tumour xenograft- mouse.	Supressed proliferation Cell-Cycle Arrest Induced Apoptosis Reduced tumour size (*in vivo*)	Inhibition of mitochondrial metabolic function and reduction of TCA cycle expression in GBM stem cells.	Dong et al.^82^
*CLOCK* Knockdown	Human GSCs.	Supressed proliferation Reduced microglia infiltration Reduced tumour growth (*in vivo*)	Triggered pro-tumour immunity via transcriptional upregulation of OLFML3, a novel chemokine recruiting immune-suppressive microglia into the tumour microenvironment	Chen et al.^83^
Adenovirus-mediated ectopic expression of *BMAL1*	U87MG cells	Supressed proliferation, migration, and invasion of glioblastoma cells.	Downregulation of Cyclin B1, Phospho-AKT, and Metalloproteinase-9	Gwon et al., 2020^84^
KL001, SHP656	Human GSCs. Intracranial tumour xenograft- mouse.	Supressed proliferation Cell-Cycle Arrest Induced Apoptosis Reduced tumour size (SHP656 in combination with SR9011, *in vivo*)	Inhibition of *Bmal1* transcription	Dong et al.^82^
SR9011, SR9009	Human GSCs.	Supressed proliferation Cell-Cycle Arrest Induce Apoptosis (Synergistic effect in combination with KL001)	Downregulation of *BMAL1* and metabolism alteration in GSCs	Dong et al.^82^
SR9011, SR9009	Human GSCs. Patient-derived GBM xenograft-mouse.	Reduced proliferation Induced apoptosis Impaired GBM growth *in vivo* and improved survival without causing overt toxicity in mice	Downregulation of autophagy and *de novo* lipogenesis	Sulli et al.^92^
SR9009	Human GSCs. Intracranial tumour xenograft- mouse.	Supressed proliferation Reduced microglia infiltration Reduced tumour growth (*in vivo*)	Downregulation of BMAL1-CLOCK complex mediated transcriptional regulation of microglia-attracting chemokine, OLFML3	Chen et al.^83^

Thus, based on the published work mentioned above, the circadian clock plays a relevant role in glioblastoma development, and hence, it may be worthy to explore the compounds mentioned, which can target clock elements and have an effect on different steps of glioblastoma development, as potential therapeutic targets.

## Perspectives

The importance of circadian rhythms in maintaining health is becoming clearer in recent years. Chronotherapy, an approach which uses the knowledge on circadian rhythms of the patients to optimize the administration time of existing treatments and thus maximizing their efficacy and/or minimizing their adverse effects, has shown promising results in different cancer types. GBM remains a deadly disease, despite the progress made in its treatment in recent years. Clinical studies on the chronomodulated administration of TMZ have shown inconclusive results, possibly due to the small number of participants and the structure of cohorts employed. Furthermore, chronotherapy regimen in the application of radiotherapy found no benefit in both extending patient survival or reducing adverse effects. Some of these studies might suffer from the retrospective character, with a rather small number of participants, and a limited influence on the cohort structure. Moreover, care must be taken in the design of chronotherapy studies, and also consider age, sex, genetic background as confounding factors. Further studies are needed to additionally determine novel genetic confounding factors which might influence the metabolism of applied drugs.

In addition to circadian, ultradian and infradian rhythms, might play a role in the response to treatments.^108^ Studies on mice with GBM showed promising results when the treatments were applied according to the metronomic 6d schedule (treatment administration every 6 days).^109^^,^^110^ Moreover, a schedule including a three-weekly^111^ or a biweekly^112^ administration of fotemustine, a third generation chloroethylnitrosourea, was explored as a second line treatment for patients with GBM recurrence, with an improved overall and progression free survival in a subset of patients.^112^ These results point to the need of considering also other rhythms in the treatment of GBM.

An additional extremely relevant point relates to the CR of the patient, it would be beneficial to explore the effect of different CR on the treatment effect. However, considering the patient's individual CR adds challenges to the administration of personalized time-dependent treatments. These include measuring personal parameters and estimating the optimal treatment timing, as well administering the treatment at the optimal time. Some of these challenges might be mitigated for some cancers by using medications in form of tablets, which can be administered at home without the supervision of the clinical staff. Moreover, simple CR measurements based on non-invasive methods, such as saliva sampling^113^ would also contribute to simplifying the routine usage of personalized chronotherapy approaches in the clinical setting. Certainly, the logistics associated to the treatment will by more complex, when additional factors like the individual CR are considered. Still, if benefits for the patient are expected to result from such a personalization, it is definitely necessary to consider it. More human studies are also needed to explore the benefits of chronomodulated application of bortezomib, 1A-116, curcumin and VX-745. Moreover, the disruption of the expression of core clock elements in GBM is well known. As such, core clock genes present an interesting target for the development of drugs that modulate circadian rhythm in GBM cells.

Entrainment of the circadian clock can be achieved also through different external zeitgebers (external agents used to synchronize the clock), such as light exposure and eating. A study on *Drosophila* has shown that re-adjustment of the light/dark cycles delayed GBM progression.^114^ In rats with glioma, constant light conditions resulted in increased tumour volume, compared to the light/dark conditions.^98^ Moreover, it is also known that eating can act as a zeitgeber and lead to circadian entrainment through an increase in the levels of plasma glucose and insulin.^115^ Tumour cells rely heavily on glycolysis to meet energy demands, leading to the higher glucose consumption.^115^ Also, GBM cells showed increased expression of insulin receptors and insulin-like growth factor receptors (IGF1R) expression,^116^ suggesting that interventions in feeding schedules may be beneficial and could be explored in the treatment of GBM. Glucocorticoids, another known zeitgeber, are frequently used in the treatment of inflammation in cancer patients. In particular, dexamethasone is used in the treatment of GBM patients. In mouse models, dexamethasone was shown to reset the circadian time in peripheral tissues.^117^ Moreover, a glucocorticoid, corticosterone, was shown to entrain the expression of *PER1* gene in the rat microglia.^118^ Altogether, these findings suggest that interventions using zeitgebers like light exposure or eating schedules, as well as timed administration of dexamethasone, may represent promising additional treatment approaches for patients with GBM.

## Conclusion and outstanding questions

With the recent advances in circadian research, in particular the new insights into the connection between the circadian clock and the molecular pathways regulating different physiological and pathophysiological processes, the expectations for disease prevention and treatment by targeting circadian rhythms have increased. As mentioned above, the dysregulation of circadian rhythms is associated with cancer onset and progression. GBM, which is considered one of the most aggressive brain tumours resistant to conventional therapies with bad prognosis, may benefit from circadian consideration in treatment regimes.

Healthy cells have a unique CR, which is mostly different than the CR of cancer cells, and therapeutic strategies targeting the circadian clock show promising results in GBM treatment. However, care must be taken when targeting molecular clock components. Many chronotherapeutic interventions targeting specific clock components have not considered the modulation effect for other components of the circadian clock machinery and how it may affect the cellular circadian rhythm. As mentioned above, some clock genes are transcription factors, such as *BMAL1* and *CLOCK*, which have variable expression levels in different GBM cells. This suggests different mechanisms affecting the transcriptional activity and the role of circadian genes in GBM progression. Thus, it will be important to understand whether those interventions are limited to targeting the circadian clock components, or if they would also affect their direct targets. Would such treatment impact other clock pathways or regulate the expression of other genes that may aggravate or alleviate tumour progression?

Despite the motivating results from the chronotherapy studies *in vitro* and on animal models that target the molecular clock in GBM, there is a low number of clinical trials in the field and further studies are needed to establish different delivery protocols in the context of GBM. Such protocols should be based on the circadian rhythm for any new anti-GBM drug or adjuvant (e.g., REV-ERB and CRY agonists, melatonin or curcumin, together with, or as a substitute to already approved TMZ), and the patient's circadian clock during the clinical treatment to improve the disease prognosis. Implications of the circadian clock in the regulation of BBB permeability will open promising opportunities for chronotherapy future in GBM. It will be interesting for the future studies to target the clock machinery affecting BBB permeability in combination with anticancer and/or immunotherapeutic agents to enhance optimal drug delivery to cancer cells.

**Search strategy and selection criteria**

The literature search was performed on clinicaltrial.gov and PubMed. The search on clinicaltrials.gov with search terms “chronotherapy” and “glioblastoma” resulted in one study. The search on PubMed using following expression was performed*: ((glioblastoma) OR (high-grade glioma) OR (high grade glioma) OR (grade 4 glioma) OR (grade IV glioma) OR (glioma) OR (low-grade glioma)) AND ((chronotherapy) OR (circadian medicine) OR (chronopharmacotherapy) OR (chronopharmaco∗) OR (chrono-chemo∗) OR (chronochemo∗) OR (chrono-radio∗) OR (chronoradio∗) OR (chronomodulated chemotherapy))*, resulting in 52 articles. Their abstracts were screened, and 10 articles were selected for the further in-depth review.

## Contributors

M.P.: literature review, analysis, visualization and figure design, writing - original draft, writing review & editing, M.H.: literature, visualization and figure design, writing - original draft, writing review & editing; O.H. literature, writing - review & editing, A.R.: conceptualization, supervision, funding acquisition, writing - original draft, writing - review & editing. All authors approved the final version of this manuscript.

## Declaration of interests

The authors declare that the research was conducted in the absence of any commercial or financial relationships that could be construed as a potential conflict of interest.
